# HTLV-1 HBZ activates mTOR signaling pathway via inhibition of GADD34

**DOI:** 10.1186/1742-4690-8-S1-A155

**Published:** 2011-06-06

**Authors:** Risa Mukai, Takayuki Ohshima

**Affiliations:** 1 Tokushima Bunri University, Sanuki, Kagawa, 769-2193, Japan

## 

Human T-cell leukemia virus type-1 (HTLV-1) infection causes adult T-cell leukemia (ATL). Recently, a novel viral protein HTLV-1 bZIP factor (HBZ), which is encoded an antisense viral gene, has been identified. HBZ may have a functional key player in cellular leukemogenesis, but the function in cells is poorly understood. To characterize the function(s) of HBZ, we performed yeast two-hybrid screen using HBZ as bait and identified growth arrest and DNA damage gene 34 (GADD34). GADD34 is induced by endoplasmic reticulum (ER) stress and the impairment of DNA. Both endogenous HBZ and GADD34 could interact in HTLV-1 infected cells. HBZ interacts with C-terminal region of GADD34 via its N-terminal region in mammalian cells. HBZ and GADD34 showed the same subcellular localization. GADD34 promotes the dephosphorylation of the factors which are downstream of mammalian target of rapamycin (mTOR), therefore, GADD34 represses mTOR activity. Our current study is focused on understanding the molecular mechanisms by which the interaction between GADD34 and HBZ regulates the mTOR signaling pathway.

